# Society Meetings

**Published:** 1897-11

**Authors:** 


### Society Meetings.

THE Medical Association of Central New York held its thirtieth annual meeting at Buffalo, Tuesday, October 19, 1897. The president, Dr. Edward B. Angell, of Rochester, took the chair at 10 o'clock and appointed the following committees: Reception, Drs. W. W. Potter, C. G. Stockton, W. C. Krauss, Edward Clark, F. C. Gram; business, Drs. Lucien Howe, John O. Roe, J. P. Creveling. He then delivered his annual address, entitled, The Hygiene of the Mind, published in full in this issue of the JOURNAL. The reading of scientific papers was continued until 1 o'clock P. M., when luncheon was served at the Ellicott Club, the members of the Association becoming the guests of the Medical Society of the County of Erie at this function. At 3 o'clock the reading of scientific papers was resumed and continued until 6 o'clock P. M. Dinner was served at the Iroquois to those remaining until evening, forty covers having been laid. Dr. J. W. Connellan, of Portland, Me., attended the meeting as an invited guest.

This was one of the most interesting and successful meetings in the history of the Association, and the proceedings, together with the papers read, will be published in the JOURNAL. The election of officers for the ensuing year resulted as follows: President, Dr. William C. Krauss, of Buffalo; vice-presidents, Dr. E. L. Mooney, of Syracuse, and Dr. E. S. Foreman, of Auburn; secretary, Dr. Charles A. Vander Beek, of Rochester, and treasurer, Dr. S. H. Elsner, of Rochester. The next meeting will be held at Auburn on the third Tuesday of October, 1898.

---

THE Mississippi Valley Medical Association held its twenty-third annual session at Louisville, Ky., October 4–7, 1897. Governor Bradley delivered the address of welcome on the part of the state and city. Dr. William H. Bailey responded in behalf of the medical profession of Louisville, thanking the governor for his cordial words of welcome. Dr. H. H. Grant, chairman of the committee of arrangements, announced the entertainments; Dr. Thomas Hunt Stucky, president of the Association, then delivered his annual address. The official badge of the Association was a unique design in oxydised sterling silver, in which was cast a portrait of Dr. Ephraim McDowell, of Danville. The address in medicine was delivered by Dr. John B. Shoemaker, of Philadelphia, and that on surgery by Dr. John B. Murphy, of Chicago. The reading of scientific papers occupied most of the time during the four days' sessions.

Among the social functions were dinners given by Drs. I. N. Bloom, T. H. Stucky, H. H. Grant, L. S. McMurtry and J. M. Mathews.

The following officers were elected for the ensuing year: President, Dr. John Young Brown, Jr., of St. Louis; vice-presidents, Dr. A. P. Buchanan, of Fort Wayne, Ind., and Dr. A. J. Oschner, of Chicago; secretary, Dr. Henry E. Tuley, of Louisville, and treasurer, Dr. Charles A. Wheaton, of St. Paul. The next meeting will be held at Nashville, on the second Tuesday in November, 1898.

---

THE Buffalo Academy of Medicine held meetings during the month of October as follows:

*Section on Surgery.*—Tuesday evening, October 5th, program: The ancestral basis of appendicitis, with specimens, by Dr. Woods Hutchinson; Report of a case of noma with recovery, by Dr. J. C. Thompson; Exhibition of cases, by Dr. Eugene A. Smith; Presentation of an apparatus for the after-treatment of suprapubic cystotomy, and exhibition of a case, by Dr. Ransom.

*Section on Obstetrics and Gynecology.*—Tuesday evening, October 12th, program: Shall we leave the uterus in situ in excision of the adnexa? by Dr. C. C. Frederick; Report of three cases of hydrocephalus, by Dr. L. Schröter.

*Section on Pathology.*—Owing to the fact that the meeting of the Medical Association of Central New York was held on this date the meeting of this section was omitted, and the members of the Academy were invited to attend the sessions of the Association.

*Section on Obstetrics and Gynecology.*—Tuesday evening, October 26th, program: Movable kidney, by Dr. Wm. G. Ring; discussion opened by Drs. Carpenter, Dowd and Congdon; Gonorrhea in women, by Dr. J. Henry Dowd.

---

THE Southern Surgical and Gynecological Association will hold its tenth annual meeting at St. Louis, November 9, 10 and 11, 1897, under the presidency of Dr. George Ben Johnston, of Richmond, Va. Dr. Johnston is a son of Senator Johnston, a nephew of General Joseph E. Johnston, and is one of the foremost surgeons in the south. The secretary, Dr. W. E. B. Davis, of Birmingham, is a leading abdominal surgeon and one of the most efficient society workers in the country. Dr. H. H. Mudd, of St. Louis, is chairman of the committee of arrangements and is laying plans for one of the most effective meetings that the Association has ever held.

